# Can the Treatment of the Insane Be Improved?

**Published:** 1895-12-14

**Authors:** 


### Can the Treatment of the Insane be Improved?

If insanity were a microbial disease it would have a much better prospect of securing for itself the careful attention of the modern scientific physician than it now has. Unfortunately, it appeals to the "clinician" mainly, and the clinician in our times is absolutely at a discount. So much the worse for medicine in general and for patients of every class in particular.

Dr. T. E. K. Stansfield, of the Claybury Asylum, insists that notwithstanding the many and great improvements which have been attained to in the general treatment of the insane, we are yet far from having reached the standard of treatment in all our institutions which science demands, and which the best experience approves. A statement of this kind, coming from such a source, calls for consideration, and, if not for approval, then for refutation. For it is a most serious reflection upon the scientific attainments and clinical capacity of the medical heads of many important lunatic asylums.

There are lunatics and lunatics, says Dr. Stansfield in effect. Many lunatics suffer from a mental derangement which is temporary, and due to causes that are temporary. With removal of the temporary causes, and treatment devoted to the toning up of the nervous system and the general health, the disease comes to an end and the patient is restored to normal sanity. As opposed to these cases there are many lunatics whose lunacy is hereditary, that is bred in them, and many others who are lunatics because of some organic change of cerebral structure. These two classes, so essentially different in character, demand different treatment and warrant a totally different prognosis. And yet, in ordinary asylums, we are told, they are all massed together in one unclassified mob, and treated, without discrimination, as permanent lunatics.

If this be indeed so, many of the responsible managers of our large lunatic asylums are much to be blamed. It was our impression that every alienist, even the youngest, was quite familiar with the principles of classification laid down by Dr. Stansfield, and that in every lunatic asylum—at any rate, in those under Imperial control—those principles of classification and the methods of treatment which naturally result therefrom were universally carried out. Those principles, we know, are carried out very largely in private practice. The ordinary physician discriminates with almost unerring accuracy between patients who are temporarily deranged in mind from temporary causes, and lunatics whose lunacy is hereditary, or is produced by structural cerebral changes. And his treatment is successful exactly in proportion as he does this. It is a revelation, by no means of a reassuring character, to be informed that in practice the officials who are responsible for care and treatment of lunatics that are numbered bythousands, are lagging behind what is well recognised by the ordinary practitioner. If we have not misinterpreted the general drift of Dr. Stansfield's report and recommendations, we are forced to the conclusion that whilst science, with unerring finger, points in the direction of a classification which will result in the happiness and cure of a large number of persons suffering from mere temporary mental derangement, asylum practice, utterly indifferent to the plain teaching of science, lumps together the temporarily deranged and the hopelessly insane in one indiscriminate mass, to the final overthrow of the reason of many who might otherwise be restored to sanity and health.

The *Morning*, commenting on Dr. Stansfield's report, urges that there is not such a radical and fundamental difference between functional and organic insanity as Dr. Stansfield insists upon. As a matter of fact, however, our lay contemporary falls into exactly the error which lay writers and perhaps some who might be expected to know better, so constantly fall into in discussing questions of physiological and pathological science. It refuses to see any facts except those of a gross and practically self-evident character. Dr. Stansfield's contentions and principles of classification are as sound in science as any demonstrated conclusions of observation and experience can possibly be. It is the manifest truth that many persons who behave like lunatics are suffering from nothing more serious than temporary mental derangement, and their cure can be effected in weeks or months. Many others, who to the outsider would present precisely the same symptoms, are hereditarily or structurally insane, and therefore, in most cases, hopeless. These two classes, so alike to the inexperienced, can nevertheless be discriminated with great facility by the competent examiner in a period of observation which can generally be measured by days.

Now this condition of things cannot be allowed to continue, at any rate in those asylums which are directly under Imperial control. It must be laid down as a rule from which there can be no deviation that in whatever direction demonstrable medical science points, in that direction medical practice must follow. In private practice this cannot always be assured, simply because private practice is private. But in Government institutions, where an adequate supervision can compel adhesion to any standard of practice which may be set up, there can be no insuperable difficulty in bringing up every department of practice in every institution to a level with whatever conclusions of science can be successfully demonstrated. We commend these facts and conclusions to those who are responsible for the administration of public lunatic asylums, and to all of every class who are interested in the important subject of the treatment of lunatics in general.

